# A comparative analysis of pairwise image stitching techniques for microscopy images

**DOI:** 10.1038/s41598-024-59626-y

**Published:** 2024-04-22

**Authors:** Fatemeh Sadat Mohammadi, Seyyed Erfan Mohammadi, Parsa Mojarad Adi, Seyed Mohammad Ali Mirkarimi, Hasti Shabani

**Affiliations:** Institute of Medical Science and Technology, Shahid Beheshti University, Tehran, Iran

**Keywords:** Microscopic images, Image stitching, Pairwise registration, Feature-based registration, Region-based registration, Biomedical engineering, Image processing

## Abstract

Stitching of microscopic images is a technique used to combine multiple overlapping images (tiles) from biological samples with a limited field of view and high resolution to create a whole slide image. Image stitching involves two main steps: pairwise registration and global alignment. Most of the computational load and the accuracy of the stitching algorithm depend on the pairwise registration method. Therefore, choosing an efficient, accurate, robust, and fast pairwise registration method is crucial in the whole slide imaging technique. This paper presents a detailed comparative analysis of different pairwise registration techniques in terms of execution time and quality. These techniques included feature-based methods such as Harris, Shi-Thomasi, FAST, ORB, BRISK, SURF, SIFT, KAZE, MSER, and deep learning-based SuperPoint features. Additionally, region-based methods were analyzed, which were based on the normalized cross-correlation (NCC) and the combination of phase correlation and NCC. Investigations have been conducted on microscopy images from different modalities such as bright-field, phase-contrast, and fluorescence. The feature-based methods were highly robust to uneven illumination in tiles. Moreover, some features were found to be more accurate and faster than region-based methods, with the SURF features identified as the most effective technique. This study provides valuable insights into the selection of the most efficient and accurate pairwise registration method for creating whole slide images, which is essential for the advancement of computational pathology and biology.

## Introduction

Whole slide imaging (WSI) is a technique that scans a whole biological sample at high resolution and combines acquired overlapping images (tiles) with the limited field of view using a stitching algorithm to generate a digital microscopic image with a wide view and high resolution. When microscopic images are stitched together, various obstacles need to be overcome. Firstly, the tiles may have a repetitive structure making mismatches in the result. Secondly, there might be empty regions in certain background tiles lacking textures, therefore providing little or no information about the overlapping regions. This makes finding a solution for these areas ill-posed. Finally, the large number of tiles involved can propagate errors in the final mosaic image and also increase the stitching time. Typical image stitching algorithms consist of two main steps: (1) pairwise registration which computes transformation between adjacent tiles, and (2) global alignment which reduces the error propagation in the mosaic image. The computational load of the image stitching is mainly allocated to the pairwise registration step. Therefore, the efficiency of the pairwise registration approach significantly affects stitching algorithm performance. Stitching algorithms can be classified into two categories based on pairwise registration: region-based and feature-based algorithms. Region-based methods calculate the similarity of pixel intensities to register tiles, while feature-based methods use salient features to compute transformation between tiles. All methods have some advantages and disadvantages. Several papers are available on comparing pairwise stitching methods in computer vision. Karami et al. compared SIFT, SURF, and ORB robustness against different transformations and deformations such as scaling, rotation, noise, fish-eye distortion, and shearing^1^. Tareen et al. also evaluated SIFT, SURF, KAZE, AKAZE, ORB, and BRISK features invariance to scale, rotation, and viewpoint^2^. Bonny et al. investigated the performance of SURF, FAST, Harris, and MSER against translation, rotation, and scaling image transformations^3^. In another work by Bonny et al.^4^, they compare correlation-based and feature-based methods and consider only a tile pair of bright-field microscopy in addition to computer vision applications. Megha et al.^5^ compare cross-correlation, phase correlation, Harris, SIFT, SURF, FAST, and ORB. Sharma et al.^6^ compare the combination of different detectors and descriptors including AGAST, AKAZE, BRISK, FAST, GFTT, KAZE, MSD, MSER, SIFT, Star, and SURF detectors, along with AKAZE, BRIEF, BRISK, DAISY, FREAK KAZE, ORB, SIFT, and SURF descriptors. However, there is no investigation on comparing pairwise stitching methods in the field of microscopic images based on our knowledge, which is very crucial in the microscopic examination of tissues and biological samples. In this paper, we compared the performance of different feature-based and region-based pairwise registration methods on microscopic images from various modalities such as bright-field, phase-contrast, and fluorescence. The feature-based methods such as Harris^7^, Shi-Thomas^8^, FAST^9^, ORB^10^, BRISK^11^, SURF^12^, SIFT^13^, KAZE^14^, MSER^15^, and deep learning-based SuperPoint features^16^ in addition to region-based methods, based on the normalized cross-correlation (NCC) and combination of phase correlation and NCC has been investigated in terms of execution time and accuracy.

## Materials and method

### Materials

**Figure 1:**
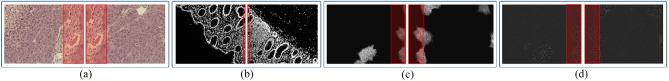
Examples of a pair of tiles in the west direction from (**a**) corrected 49–01 bright-field image set from the Tak dataset, (**b**) fluorescence image set from the human colon dataset, (**c**) fluorescence image set from the stem cell colony dataset, and (**d**) phase-contrast image set from stem cell colony dataset.

We utilized various experimental microscopy datasets that differed in modality, number of tiles, and overlaps to evaluate the pairwise stitching algorithms: (1) the collection of bright-field images prepared by Tak et al.^17^ consisting of ten samples of different cells with different densities. Each sample contains 100 tiles in the form of a 10 × 10 grid. This collection contains two sets of tiles for each sample: the set of tiles that was the direct output of the light microscope with no pre-processing having shading and fixed-pattern noise, and the set of tiles that were corrected using a golden standard uneven-illumination correction method, Empty-Zero algorithm^18^, which is one of the most important steps in creating whole-slide images, though not the purpose of this study. In this study, both sets were used to evaluate different approaches. (2) An image collection of a human normal colon sample^19^ from fluorescence modality, featuring small overlapping areas between tiles. (3) The collection of stem cell colony dataset images^20^ from the phase-contrast and fluorescence modalities. Table 1 provides a summary of the image datasets used in this study, and Fig. 1 illustrates example tile pairs from the experimental datasets.

**Table 1:** Detailed overview of the microscopic image datasets used in this study.

Dataset	Modality	Samples	Tiles (grid)	Total tile pairs used in pairwise registration	Overlap	Magnification	Size of tile
Tak^17^	Bright-field	10	100 (10 × 10)	1800	25%	40 ×	2304 × 1719
Human colon^19^	Fluorescence	1	609 (29 × 21)	1168	2–3%	20 ×	1280 × 1080
Stem Cell colony^20^	Fluorescence	3	552 (23 × 24), 100 (10 × 10), 25 (5 × 5)	1057, 180, 40	10%, 10%, 19%	10 ×	1392 × 1040
	Phase-contrast	2, 1	100 (10 × 10), 25 (5 × 5)	360, 40	10%, 19%

We sorted the image collection of each dataset based on their scanning pattern to perform different pairwise registration methods on each tile with its adjacent north and west neighbors. For image collection in the form of a M × N grid, the number of transformations computed in the pairwise registration is 2MN-(M + N), which is summarized in Table 1 for each dataset. To assess the performance of each method, we selected tile pairs that contain texture rather than background in their overlapping region. For example, for the Tak dataset which includes 1800 tile pairs, 1603 tile pairs meet this criterion. Similarly, 852 out of 1168 tile pairs for the human colon dataset, 351 out of 1067 tile pairs, 61 out of 180 tile pairs, and 21 out of 40 tile pairs for the stem cell colony dataset with fluorescence modality, and all tiles for the stem cell colony dataset with the phase-contrast modality meet the criterion.

### Method

**Figure 2:**
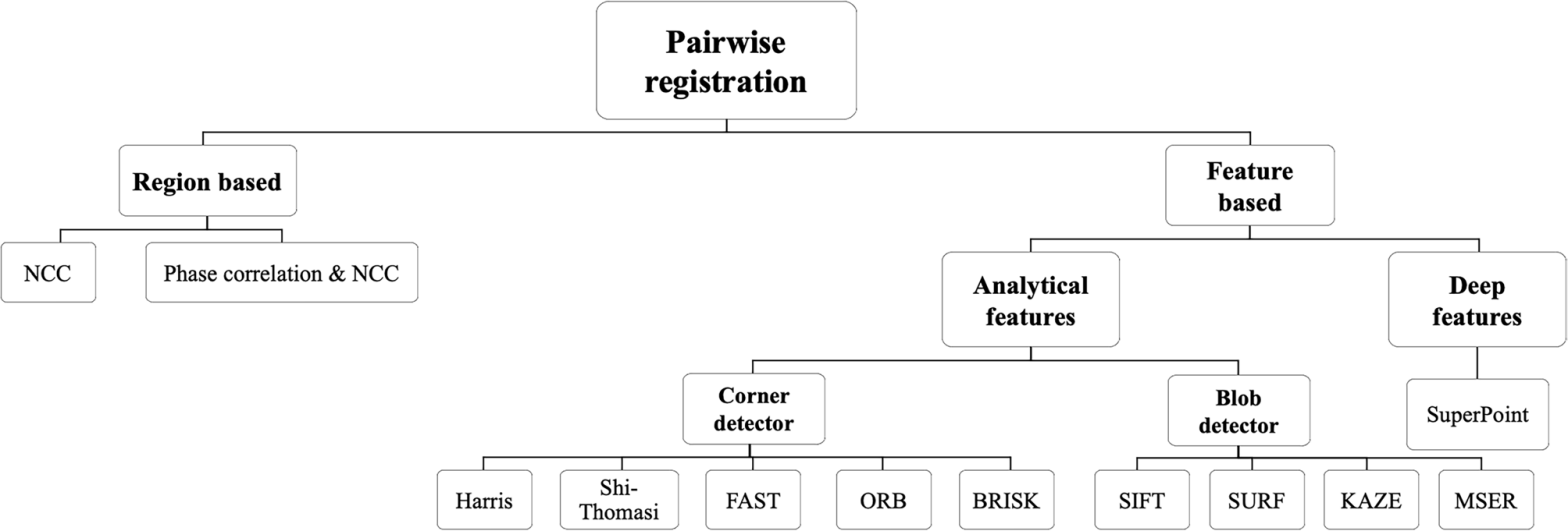
Different types of pairwise registration methods.

In the WSI technique, the scanning pattern reveals the order of connection between tiles, which is used to sort the tiles in a specific and predetermined pattern. Therefore, we focused on computing the transformation of each tile based on its adjacent north and west tiles in the pairwise registration step. The transformation model used in this study was assumed transitional. The model ignored scaling and rotation parameters and only estimated displacement parameters in the horizontal and vertical directions. This is because in the WSI technique, the sample was placed under the objective lens at a fixed angle with negligible changes, and it was imaged at a constant magnification by moving the microscope motors in horizontal and vertical directions. Robust and efficient pairwise registration makes the stitching algorithm perform fast with high accuracy. Pairwise registration approaches can be categorized into two groups: feature-based and region-based. Figure 2 shows different types of pairwise registration methods.

#### Feature-based

Feature-based registration methods compute the pairwise transformation using sparse feature points which consist of four main steps: feature detection and description, feature matching, outlier rejection, and transformation computation. The first step involves detecting and describing features in the overlapping region of the two tiles that identify key points such as corners, blobs, or deep features. It is important to note that the orientation of feature vector descriptors was not estimated. We evaluated multiple approaches for feature detection, including analytical methods^7–15^ and Self-Supervised Interest Point (SuperPoint)^16^, which is based on deep learning for feature detection and description.

Analytical feature detection methods are classified into two categories: corner detectors and blob detectors. Corners arise from the intersection of two lines where there is a sharp difference in brightness. The Harris corner detector introduced by Harris and Stephen in 1988^7^, detects corners by shifting the Gaussian window in all directions and measuring differences in the intensity. Later in 1994, Shi and Tomasi improved the corner selection criteria of the Harris corner detector to achieve better performance^8^. In 2006, Rosten and Drummond proposed the Features from Accelerated Segment Test (FAST) corner detector^9^. This algorithm is computationally efficient and suitable for real-time applications but does not compute the orientation. In 2011, Rublee et al. presented the Oriented FAST and Rotated BRIEF (ORB) algorithm, which is a fusion of a modified FAST feature detector and BRIEF (Binary Robust Independent Elementary Features) descriptor^10^. This algorithm makes the feature points invariant to rotation. Also, in 2011, Leutenegger et al. introduced the Binary Robust Invariant Scalable Keypoints (BRISK) corner detector which is invariant to scale and rotation^11^.

Blob detectors, on the other hand, identify regions in the image that differ in properties such as brightness, color, or texture from surrounding regions. One of the popular blob detectors is the Scale Invariant Feature Transform (SIFT) introduced by Lowe in 2004^13^. The SIFT algorithm detects feature points by selecting scale-space extrema of the difference of Gaussians. Although SIFT is scale and rotation-invariant, it is computationally expensive. To overcome this issue, the Speeded-Up Robust Features (SURF) was introduced in 2006^12^ which is a faster version of SIFT. The SURF detector works based on the determinant of the Hessian matrix and can be used in real-time applications with a lower computational cost than SIFT. In 2012, KAZE features were proposed by Alcantarilla et al.^14^. These features are invariant to rotation and scale and have more distinctiveness at varying scales with the cost of a moderate increase in computational time. Another algorithm, Maximally Stable Extremal Regions (MSER), was proposed by Metas et al. in 2002^15^. The MSER algorithm detects blob-like structures by identifying regions that remain stable over various thresholds.

Moreover, we investigated a self-supervised method known as SuperPoint^16^. This method employs a fully convolutional neural network to detect and describe interest points. In this study, a pre-trained model available on the SuperPoint Github repository is used to extract features and descriptors.

After the feature detection-description, the next is the feature matching step to find corresponding features between the two images using the nearest neighbor distance ratio. We utilized the Brute-force matching algorithm. The feature distance is computed using the sum of squared differences for general features or Hamming distance for binary features. However, the feature-matching process can often result in incorrect matches. The third step is outlier rejection to filter out incorrect matched points. This step is important as the transformation parameters are computed based on inlier features and significantly affect the performance. To accomplish this, we applied the probabilistic M-estimator Sample Consensus (MSAC) algorithm^21^ with 2000 iterations and 99.99% confidence. This algorithm removes the outliers and fits the transformation model (Homography Matrix) parameters based on inlier features. However, for SuperPoint features, we utilized the Random Sample Consensus (RANSAC) algorithm^22^ to remove the outliers and estimate the translation parameters between two tiles, as the code is executed in Python.

#### Region-based

The region-based method computes the transformation between two pairs with shifting windows of the reference template relative to the target image and compares the similarity of pixels using different criteria. In this study, we have used two approaches, NCC and a combination of phase correlation and NCC. To implement NCC, the window of the overlapping region of the source tile is shifted over the search region in the target tile, and displacement with the maximum NCC is determined. The search region is defined as the overlapping region of the target tile + 10% of the width/height of tiles to account for imprecise scans and provide an error margin according to^23^. The phase correlation method employs the Fourier transform to compute phase correlation in the frequency domain. The resulting phase correlation peaks are used to select four translations, and the transformation with maximum NCC is ultimately chosen. To implement pairwise registration using phase correlation and NCC we utilized the pairwise registration part of the Microscopy Image Stitching Tool (MIST) source code^24^.

### Evaluation metrics

To assess the effectiveness of various pairwise registration techniques, we analyzed the time required for pairwise registration and the root mean square error (RMSE) of the pixel intensity within the overlapping regions of adjacent tiles. We provide additional metrics such as the number of extracted feature points, the number of matched feature points, and the number of inlier feature points for feature-based methods. Furthermore, the number of failed attempts to compute the translation parameters referred to as “not-found translations”, and invalid translations which is out of the valid range. The valid translation range is obtained by overlapping region of tiles ± 2% of the width/height of tiles, accounting for uncertainty due to the imperfections of the automated microscope stages. This comprehensive evaluation allows us to make informed decisions regarding the most appropriate pairwise registration method for a given application.

## Results

The experiments were performed using MATLAB 2023a software on an Intel® Core™ i7-1165G7 CPU @ 2.80 GHz operating system with 16 GB of memory. Due to the high computational demand of the SuperPoint codes, execution on the same operating system that was used for other approaches was very time-consuming. Therefore, the SuperPoint codes were also executed on Google Colab, a cloud-based platform that provides free access to GPU resources with 2 Intel Xeon CPUs @ 2.20 GHz, 13 GB of RAM, and a NVIDIA T4 GPU with 12 GB of VRAM.

It is worth mentioning that all hyperparameters for analytical feature-based approaches are set to the default values based on MATLAB implementation. To determine the best hyperparameters for the deep learning-based SuperPoint features, we performed a greedy search using the 234–01-67 corrected image collection of the Tak dataset and chose the best value of different hyperparameters that resulted in the lowest execution time and RMSE. The optimal configuration for the SuperPoint model was set to a threshold of 0.001, patch size of 512, batch size of 8, and overlap of 0.8.

The first investigation is done using the Tak dataset which is less challenging compared to the other two datasets (Table 1), because the bright-field modality provides rich structures of tissues and biological samples in comparison with the fluorescence and the phase-contrast modalities. Moreover, the amount of overlap of the Tak dataset (25%) is not as critical as the Human colon dataset (2–3%). Furthermore, the Tak dataset provided the original tiles (with shading pattern) and corrected tiles (without shading pattern) of samples which we can use them to check the robustness of different methods to illumination variations.

**Figure 3:**
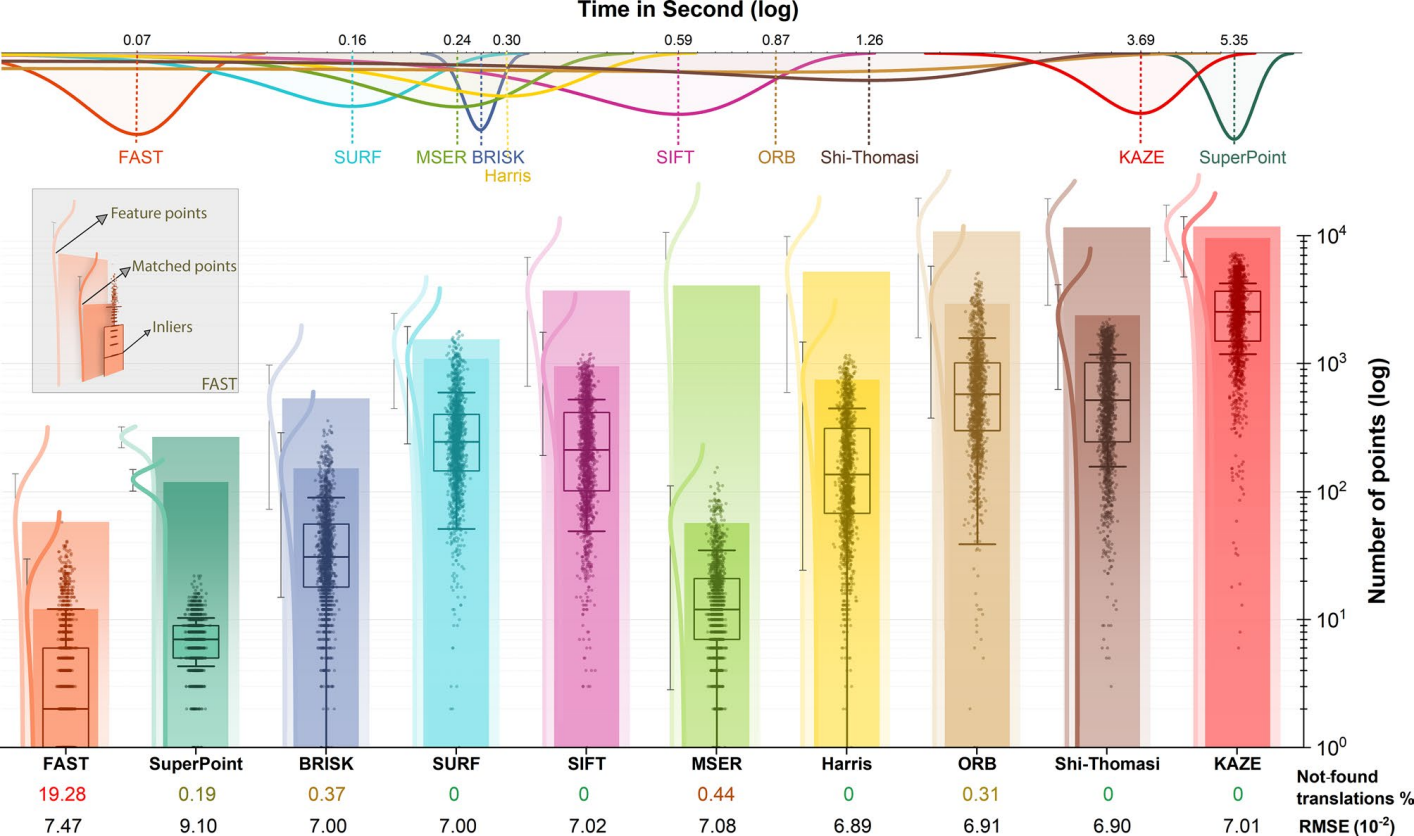
Number of detected, matched, and inlier feature points, percentage of failures in computing transformation, distribution of execution time, and the RMSE of 1603 textured pair tiles from corrected image collections of the Tak dataset using feature-based pairwise registration methods considering the overlapping region for feature extraction.

The results of feature-based pairwise registration using 1603 textured tile pairs (described in the Materials section) of the corrected collection of the Tak dataset, considering the overlapping region for feature extraction, have been summarized in Fig. 3 and Table 2. Figure 3 includes the distribution of the required time to compute the translation parameters between the adjacent tiles, the RMSE, the number of extracted feature points, the number of matched feature points, the number of inlier feature points, and the percentage of not-found translations. It is obvious that there is no relation between the number of feature points and the execution time. The results indicate that the SURF, SIFT, Harris, Shi-Thomasi, and KAZE methods successfully computed translations for all pair tiles including texture. In contrast, the FAST method had the highest percentage of failed attempts to compute translations at 19.28% which means it could not compute translations for 309 of 1603 pair tiles. After FAST, it has been followed by MSER at 0.44%, BRISK at 0.37%, ORB at 0.31%, and SuperPoint at 0.19%. The processing time of the FAST method was found to be shorter than other methods and the SURF, MSER, BRISK, Harris, SIFT, ORB, Shi-Thomasi, KAZE, and SuperPoint are ordered in an increasing manner. Despite using Google Colab, SuperPoint’s execution time is higher than other methods except the KAZE method. It is worth mentioning that the ORB had a long execution time on the 53–03 image collection of the Tak dataset due to the dense textures present in tiles. This makes the distribution of the ORB execution time very flat. Regarding the RMSE values, Harris had the lowest value, followed by Shi-Thomasi, ORB, BRISK, SURF, KAZE, SIFT, MSER, FAST, and SuperPoint. However, the RMSE values of different approaches except the SuperPoint are very close together, and the statistical analysis is reported in Table 2. The number of extracted features was highest for KAZE, followed by Shi-Thomasi, ORB, Harris, MSER, SIFT, SURF, BRISK, SuperPoint, and FAST. In contrast, the number of matched features was highest for KAZE, ORB, Shi-Thomasi, SURF, SIFT, Harris, BRISK, SuperPoint, MSER, and FAST. Finally, the number of inlier features was highest for KAZE, ORB, Shi-Thomasi, SURF, SIFT, Harris, BRISK, MSER, SuperPoint, and FAST.

**Table 2:** The RMSE, execution time, number of detected, matched, and inlier feature points, number of not-found translations, and the number of invalid translations of 1603 textured pair tiles from image collections of the Tak dataset using region-based and feature-based pairwise registration methods considering the overlapping region for feature extraction. Note that the mean ± std is reported and a ranked ANOVA test for RMSE of corrected images is F(11, 18,894) = 139.77, *p* = 0.0 < 0.001.

Dataset	Method	RMSE (10^–2^)	Time (second)	Feature points	Matched points	Inliers	Not-found translations	Invalid translations
Tak, corrected images	PhaseNCC	7.28 ± 2.77	0.49 ± 0.02	–	–	–	0	99
	NCC	6.66 ± 1.82	0.92 ± 0.07	–	–	–	0	0
	FAST	7.57 ± 2.17	0.07 ± 0.01	58 ± 79	12 ± 17	5 ± 7	309	3
	SuperPoint	9.10 ± 2.48	5.35 ± 0.43	269 ± 49	119 ± 23	7 ± 3	3	3
	BRISK	7.00 ± 2.03	0.27 ± 0.08	535 ± 460	153 ± 138	45 ± 45	6	0
	SURF	7.00 ± 2.10	0.16 ± 0.05	1554 ± 1080	1092 ± 856	324 ± 273	0	0
	SIFT	7.02 ± 2.10	0.59 ± 0.21	3743 ± 3077	958 ± 769	288 ± 238	0	0
	MSER	7.08 ± 2.05	0.24 ± 0.08	4107 ± 6566	57 ± 54	18 ± 17	7	0
	Harris	6.89 ± 2.08	0.30 ± 0.10	5251 ± 4654	752 ± 728	224 ± 224	0	1
	ORB	6.91 ± 2.03	0.87 ± 1.29	10,860 ± 9234	2953 ± 2595	813 ± 774	5	0
	Shi-Thomasi	6.90 ± 2.06	1.26 ± 0.79	11,663 ± 8682	2389 ± 1760	669 ± 512	0	0
	KAZE	7.01 ± 2.09	3.69 ± 0.65	11,844 ± 5522	9664 ± 4793	2727 ± 1537	0	0
Tak, images with shading	PhaseNCC	20.92 ± 11.85	0.49 ± 0.02	–	–	–	0	1411
	NCC	16.74 ± 6.54	0.94 ± 0.08	–	–	–	0	168
	FAST	16.05 ± 6.07	0.07 ± 0.01	72 ± 100	11 ± 17	5 ± 7	363	3
	SuperPoint	21.04 ± 8.88	5.04 ± 0.26	269 ± 50	119 ± 23	7 ± 3	0	3
	BRISK	17.41 ± 7.46	0.27 ± 0.02	592 ± 587	118 ± 126	37 ± 39	15	0
	SURF	17.47 ± 7.46	0.16 ± 0.05	1609 ± 1243	872 ± 779	272 ± 243	1	0
	SIFT	17.48 ± 7.46	0.59 ± 0.24	4761 ± 3076	733 ± 676	229 ± 208	0	0
	MSER	17.48 ± 7.47	0.26 ± 0.07	801 ± 711	43 ± 48	14 ± 15	12	0
	Harris	17.42 ± 7.50	0.30 ± 0.10	3161 ± 2697	629 ± 642	196 ± 204	1	1
	ORB	17.37 ± 7.48	0.85 ± 1.32	13,997 ± 10,848	2138 ± 2272	618 ± 645	9	0
	Shi-Thomasi	17.42 ± 7.49	1.56 ± 0.80	18,549 ± 7236	1920 ± 1447	545 ± 422	0	0
	KAZE	17.47 ± 7.46	3.53 ± 0.69	11,758 ± 5438	8597 ± 4490	2525 ± 1443	0	0

**Figure 4:**
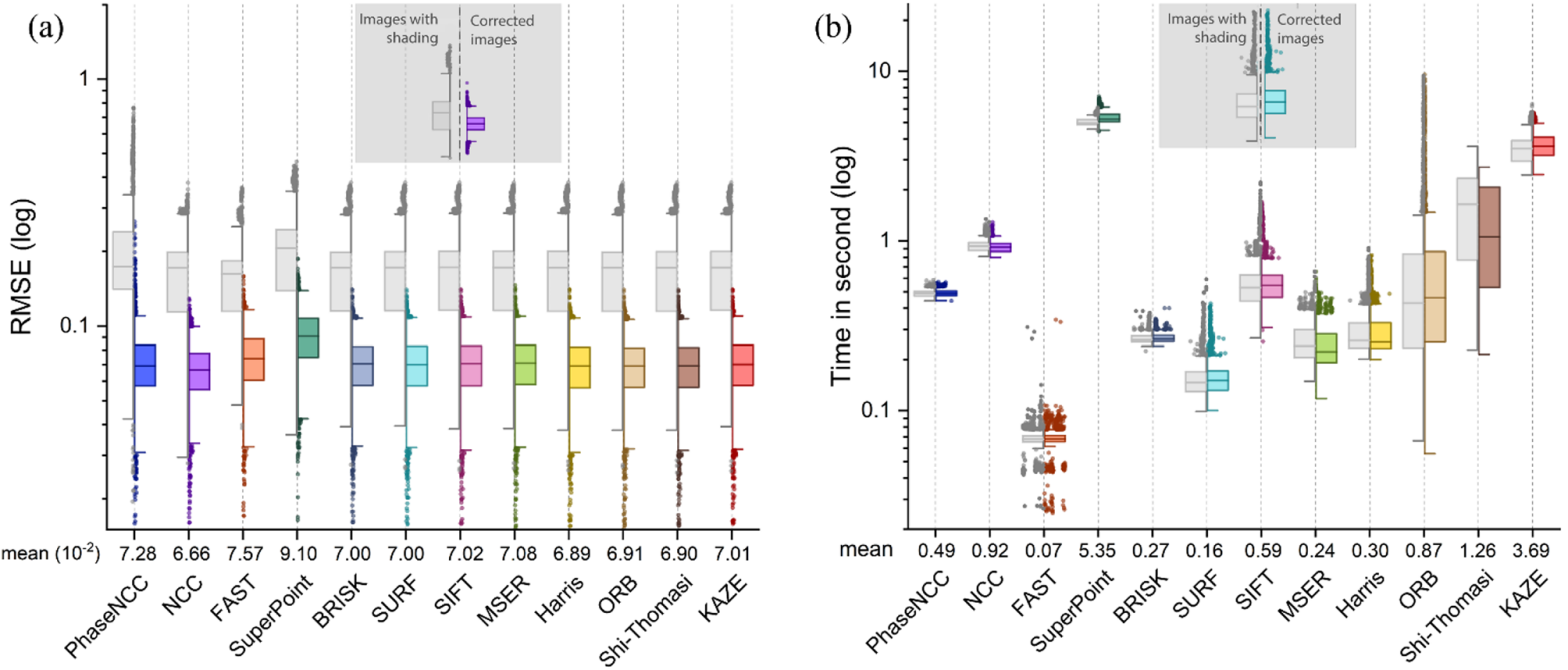
Distribution of (**a**) RMSE and (**b**) execution time of corrected and original tiles from 1603 textured tiles of the Tak dataset using region-based and feature-based pairwise registration methods considering the overlapping region for feature extraction. Note that the mean value of the RMSE is reported for corrected images.

**Figure 5:**
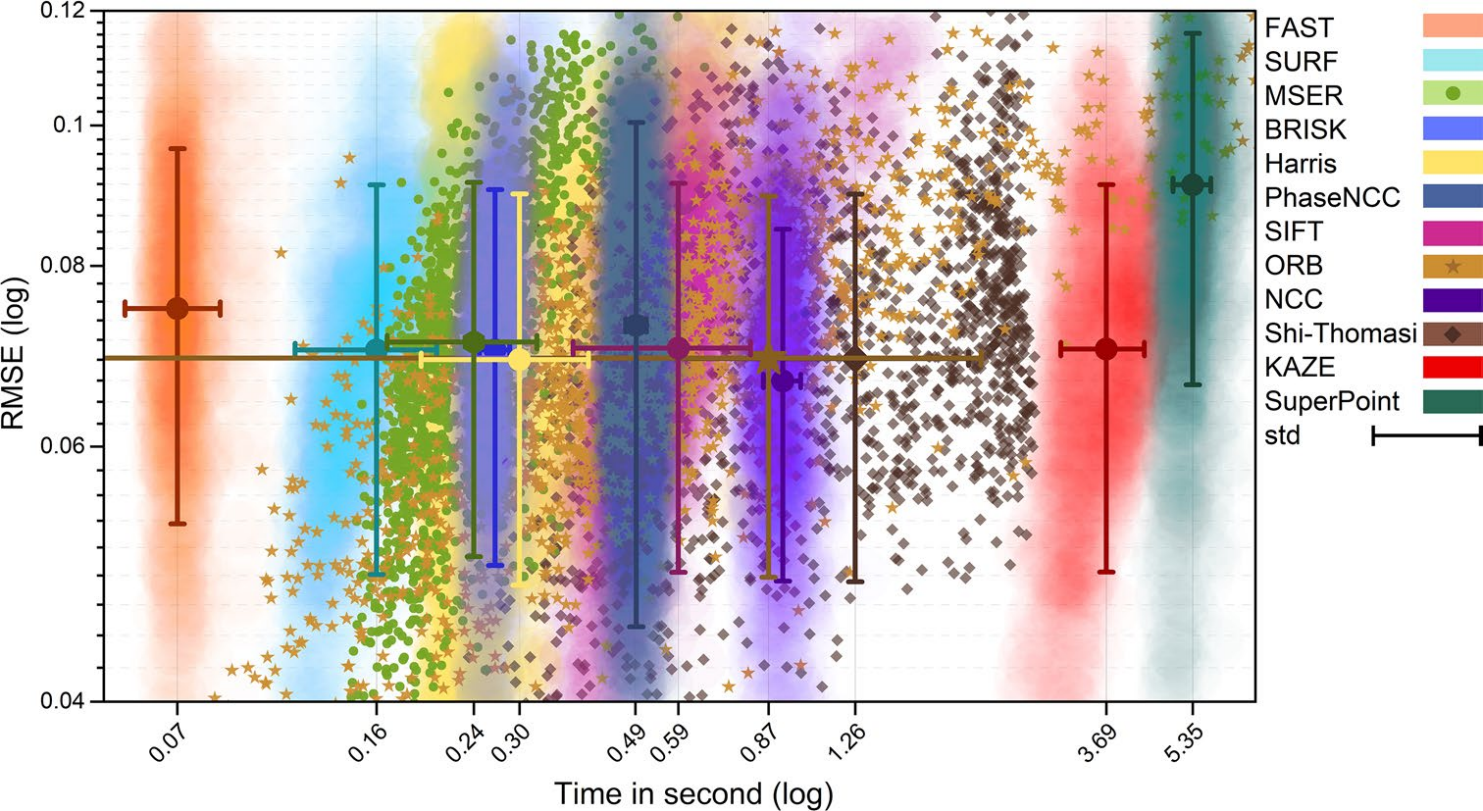
The scatter plot of the RMSE and execution time of 1603 textured tiles of the corrected Tak dataset using region-based and feature-based pairwise registration methods considering the overlapping region for feature extraction.

**Figure 6:**
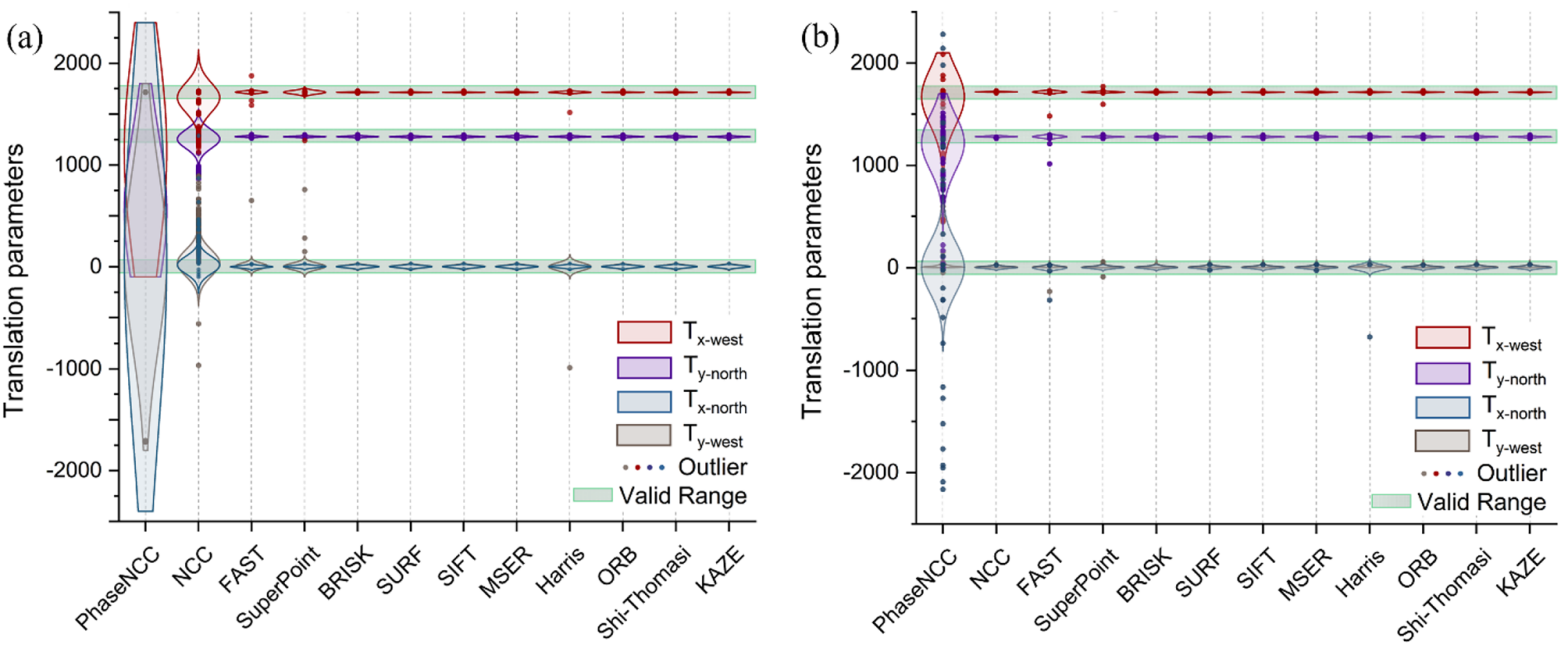
Distribution of computed translation parameters T_x_ and T_y_ in the west and north directions for (**a**) original tiles with shading and (**b**) corrected tiles without shading pattern from 1603 textured tiles of the Tak dataset using region-based and feature-based pairwise registration methods considering the overlap region for feature extraction. Note that the green boxes indicate the valid translation ranges.

To determine the superior approach among region-based and feature-based methods, various evaluation metrics were employed using the original and corrected tiles of the Tak dataset to investigate their robustness to the shading artifact which is a common problem in microscopic images, particularly in the bright-field modality. The evaluation metrics include the RMSE (Fig. 4a), execution time (Fig. 4b), the RMSE and the execution time (Fig. 5), computed translation parameters in vertical and horizontal directions, and corresponding invalid translations (Fig. 6).

In the evaluation of corrected tiles, our findings demonstrated that region-based methods performed slightly better than feature-based methods in terms of RMSE, however, the execution time of Phase-NCC and NCC is longer than that of FAST, SURF, MSER, BRISK, Harris, ORB, and SIFT. The same clarification can be obtained using the analysis of original tiles with shading except the range of the RMSE is bigger as the difference between pixel’s intensity is higher in the presence of shading.

We have depicted the scatter plot of the RMSE and the execution time in Fig. 5 to summarize the results. This plot emphasizes the wide range of execution time of MSER, ORB, and Shi-Thomasi methods which is compatible with the results reported in Table 2.

The RMSE metric is not sufficient to peak the most efficient method because the RMSE values are very close in different approaches. Additionally, the RMSE value only relies on the similarity of pixel intensities in the overlapping region of tiles and does not evaluate whether the tiles are transformed correctly or not. For this reason, we compared computed translation parameters (T_x_ and T_y_) using different pairwise registration methods with valid translation ranges. In the evaluation of corrected images, results revealed that a large number of computed translation parameters using the Phase-NCC method (6.18%) were out of the valid range. Additionally, FAST, SuperPoint, and Harris methods also had multiple translations out of range. However, the translations computed by NCC and other feature-based methods such as BRISK, SURF, SIFT, MSER, ORB, Shi-Thomasi, and KAZE were all within the valid range. The results obtained using original tiles with shading indicate that the feature-based methods outperformed region-based ones when comparing the valid translation parameters. The Phase-NCC and NCC methods computed a large number of translations outside the valid range (88.02% and 10.48%, respectively). Similarly, FAST, SuperPoint, and Harris methods had translations out of range whose number is very low compared to Phase-NCC and NCC. However, translations computed by other feature-based methods like BRISK, SURF, SIFT, MSER, ORB, Shi-Thomasi, and KAZE were all within the valid range.

All evaluation metrics, besides statistical analysis for RMSE values, are summarized in Table 2.

We also inspected the SuperPoints features in more detail to investigate whether the reason for suboptimal performance is related to the type of the features or matching algorithm. We compared the Brute-force and the LightGlue^25^ matching algorithms. Since the SuperPoints features (available in the SuperPoint GitHub repository) do not provide a score for features we could not match the SuperPoint with LightGlue. There is a new implementation of the SuperPoint (available in the LightGlue GitHub repository), we named it SuperPoint^*^ here, which is compatible and can be matched with the LightGlue matching algorithm. The results are provided in Table 3 in terms of the RMSE and time values for the SuperPoint features alongside the Brute-force matching algorithm, the SuperPoint^*^ alongside the Brute-force and the LightGlue matching algorithms. All three of them were executed on a high-end PC, with an Intel Core-i7 9700k CPU, NVIDIA RTX 3050-8GB GPU with 16 GB of RAM.

**Table 3:** The RMSE and execution time of 1603 textured pair tiles from corrected image collections of the Tak dataset using two different implementations of the SuperPoint features considering the overlapping region for feature extraction and two different matching algorithms. Note that SuperPoints is implemented using the SuperPoint GitHub repository and SuperPoint^*^ is implemented using the LightGlue GitHub repository.

Feature Extraction	Matching Algorithm	RMSE (10^–2^) (mean ± std)	Time in second (mean ± std)
SuperPoint	Brute-force	9.05 ± 2.35	2.62 ± 0.11
SuperPoint*	Brute-force	21.28 ± 5.82	0.42 ± 0.04
SuperPoint*	LightGlue	21.26 ± 5.80	0.36 ± 0.06

The results show that the effect of the matching algorithm is not critical (rows 2–3 of Table 3): the same RMSE and slightly different in execution time. However, replacing the SuperPoint with SuperPoint^*^ reduces the execution time significantly on one side and increases the RMSE on another side. This is because of different implementations and weights for the SuperPoint feature extraction technique.

## Discussion

Among various methods investigated for pairwise registration of microscopy images, the FAST method is the fastest, although it does come with the drawback of having the highest percentage of failed attempts, standing at 19.28%. Additionally, its RMSE value is higher when compared to other methods, except for SuperPoint. On the other hand, the MSER method is capable of detecting a high number of features between tiles, but it is unable to match as many features, leading to a near 100-fold decrease. Our analysis reveals that the feature-based methods outperform the region-based methods as Phase-NCC computed translation parameters out of valid translation range in both collections of the Tak dataset: corrected images and images with uneven illumination and NCC computed translation parameters out of valid translation range in original tiles with uneven illumination of the Tak dataset. In addition, there is no preference for using region-based pairwise registration based on computational time and RMSE value. Among the investigated feature-based methods, SURF, SIFT, Harris, Shi-Thomasi, and KAZE methods were all successful in computing translation parameters. However, KAZE and Shi-Thomasi methods required a higher computational time due to their high number of detected and matched features. One translation parameter computed using the Harris method was out of the valid range for the Tak dataset images with and without shading. Although the SIFT method was able to detect more features than SURF features, SIFT-matched features were lower. The SURF features also result in a lower execution time and RMSE value than SIFT. More investigations on the SuperPoint features revealed that fine-tuning the SuperPoint network for microscopic images might improve the RMSE and reach better performance. Overall, the SURF features proved to be more effective in pairwise registration of microscopic images in terms of computational time, error, and robustness to the illumination variations.

**Figure 7:**
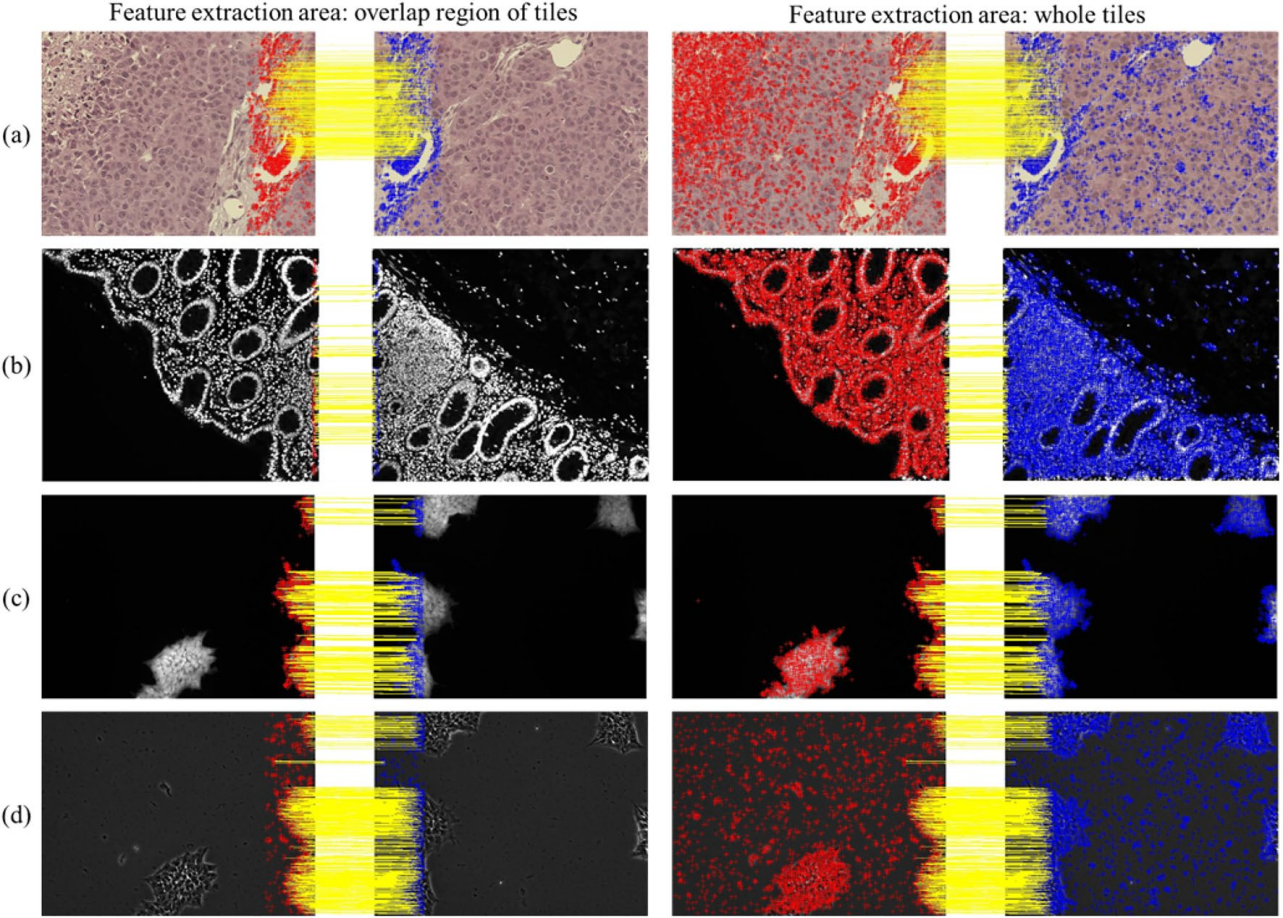
Detected feature points and inlier feature points in the overlap region and entire image of tile pairs in the west direction using the SURF feature extraction method from (**a**) corrected 49–01 bright-field image set of the Tak dataset, (**b**) fluorescence image set of the human colon dataset, (**c**) fluorescence image set of the stem cell colony dataset, and (**d**) phase-contrast image set of stem cell colony dataset.

We also analyzed the SURF features in pairwise registration of microscopic images when the location and the amount of the overlapped region are not provided and the method involves detecting and extracting features from the entire image. Our analysis based on RMSE, execution time, and translation parameters indicates that the RMSE values and translation parameters remained consistent while extending feature extraction to the whole region resulted in significantly higher execution time. One example of tile pairs from different experimental datasets is shown in Fig. 7. The figure shows the detected features and inlier feature points using the overlap region and the entire image, providing a clearer visual representation of how efficiently the SURF features work regardless of prior knowledge about the overlapping region. Our results indicate that extending the feature extraction region does not compromise the computed transformation's accuracy, as inlier points are almost the same. On the other hand, it considerably reduces the computational load, as most of the extracted feature points from the whole image were not useful in computing the translation parameters.

In addition, the SURF features are examined considering other modalities of microscopic images such as phase-contrast and fluorescence as shown in Fig. 7b-d. It should be mentioned that the SURF feature extraction method calculates the determinants of the Hessian matrix in both space and scale and eliminates points that have response values below a specific threshold. The threshold value depends on the image and the intended application. We used 1000, the default threshold value for the Tak and the human colon datasets which are rich in texture. However, we encountered a challenge in detecting SURF features in the stem cell colony dataset. When using the default threshold value of 1000, we were able to extract only a small number of features or none at all, making it impossible to compute the transformation between two tiles unless textures were present in the overlap region. To overcome this challenge, we closely examined randomly selected images to determine the number of features and Hessian determinant values and eventually determined that an optimal threshold value of 0.1 for fluorescence and 1 for phase-contrast allowed us to extract sufficient features and successfully compute tile transformations for our experiments.

We summarized the result of the SURF features for all datasets in Table 4 considering one more evaluation metric: the structured similarity matrix (SSIM). Evaluating the results of different datasets is crucial as we can see the RMSE and SSIM are better for the Stem cell colony dataset rather than the Tak and human colon dataset. This is because the texture of the Tak and human colon datasets are very dense and any difference would cause to higher RMSE and lower SSIM. We also reported invalid translations to show the robustness of the SURF features in pairwise registration, regardless of datasets.

**Table 4:** The percentage of invalid translations, RMSE, and SSIM of pairwise registration using SURF features considering the overlap region for feature extraction for all datasets used in this study.

Dataset	Invalid translations %	RMSE (10^–2^) (mean ± std)	SSIM (mean ± std)
Tak	0	7.00 ± 2.11	0.80 ± 0.08
Human colon	0.1	9.11 ± 4.02	0.91 ± 0.11
Stem Cell colony (Fluorescence)	0	0.16 ± 0.15	0.98 ± 0.04
Stem Cell colony (Phase-contrast)	0.25	0.21 ± 0.11	0.92 ± 0.06

## Conclusion

This study presents a comparison of region-based pairwise registration methods, namely NCC and Phase-NCC, with feature-based methods such as Harris, Shi-Thomasi, FAST, ORB, BRISK, SURF, SIFT, KAZE, MSER, and deep learning-based SuperPoint features. The investigation results on the experimental microscopy images reveal that feature-based methods outperformed region-based methods in terms of accuracy and processing speed. Moreover, feature-based methods were found to be highly robust to uneven illumination of tiles. Among feature-based methods, the SURF features were found to be the most effective, surpassing all other techniques on different image modalities, including bright-field, phase-contrast, and fluorescence. This study provides valuable insights into the strengths and weaknesses of different registration methods, which can be useful for researchers working in the field of microscopic image stitching.

## Data Availability

The Tak dataset which was included in the published article^17^, can be accessed at https://github.com/pair-kopti/Shading-correction. Similarly, the human colon dataset is available through Sage Synapse at 10.7303/syn25826362 (a free account is required to access the data). Additionally, the images of the stem cell colony dataset can be downloaded from https://isg.nist.gov/BII_2015/webPages/pages/stitching/Stitching.html.

## Code availability

https://github.com/labCOI/microscopy_pairwise_register.git.
